# Book Review: The Sleep Solution: Secrets for a Good Night’s Sleep

**DOI:** 10.3389/fneur.2017.00076

**Published:** 2017-03-07

**Authors:** Seithikurippu R. Pandi-Perumal

**Affiliations:** ^1^Somnogen Canada Inc., Toronto, ON, Canada

**Keywords:** sleep, sleep disorders, sleep medicine specialty, patient education, sleep loss, sleep deprivation

Sleep disorders affect not only the person with the disease but also the person’s family, friends, caregivers, society, and the population at large.

The author of this volume Dr. Manvir Bhatia was trained by premier institutes, such as the All India Institute of Medical Sciences and Harvard, and is a well-recognized sleep expert both nationally and internationally. The book’s Foreword and Afterword were also written by some of the world’s most notable sleep experts, namely, Prof. Christian Guilleminault of Stanford University and Prof. Sudhansu Chokroverty of JFK Medical Center in Edison, NJ, USA, respectively.

At the beginning of the book, the author has mentioned that the aim of this book was to serve as an all-encompassing guide to sleep-related issues for everyone, regardless of age and gender. In order to fulfill this need, this book is organized into four parts: Part I—Understanding sleep; Part II—Sleep and the body; Part III—Sleep problems; and Part IV—Solutions. Part I introduces the subject to the readers and provides a brief account on what sleep is, why we sleep, understanding sleep, how much sleep we need, effects of sleep deprivation and sleep debt, and the measurement of sleep. In part II, the author explores sleep and health, sleep and beauty, and sleep and food. In part III of this book, the most common sleep disorders are discussed, as well as other topics that will interest the general reader. These include sleep and technology, the adverse effects of technology, as well as the effects on sleep of lifestyle, substance abuse, and relationships. Finally, part IV of the book provides practical advice concerning sleep hygiene and how to obtain more restful sleep.

Readers will also benefit from simple screening tools for evaluating sleep, summary boxes, figures, tables, sample case histories, and key points for better sleep, which interspersed throughout the text. Finally, the book also provides further guidance regarding relaxation methods, sleep hygiene practices, as well as simple yoga asanas to help with sleep issues.

Dr. Bhatia’s writing style is very readable and easy to follow. The author goes into only as much detail as is needed for the reader to follow her main theses. The book is well-organized, well-balanced, wonderfully written, and provides an overview of sleep and sleep disorders in a plain and simple language. This book will appeal to both patients who have sleep disorders and physicians who treat sleep disorders alike. The book will be of interest to trainees as well as clinical sleep researchers who want to have a basic understanding of sleep.

The book is informative and fulfils an important need in many parts of the world including India where an understanding and appreciation of the importance of sleep to overall health is still in its early stages. This book will be a great read if one is looking to improve the quality of their sleep and general well-being. In addition, this book will be an important addition to one’s personal library no matter if someone is a patient or a physician.

In short, the author has done a good job explaining the basic aspects of sleep medicine and has written the book that maintains readers’ interest. Dr. Bhatia’s book is a good choice for those who want a brief introduction to the science of sleep.

This book is not a reference volume, nor does it provide depth of medical information for the sleep disorders patient or parents of children with serious sleep problems. This volume may be more appropriate for a sleep specialist to recommend to his/her patients. I highly recommend this book to anyone interested in an introduction to sleep medicine and sleep disorders and a must have for patient libraries.

## Author Contributions

The author confirms being the sole contributor of this work and approved it for publication.

## Conflict of Interest Statement

The author has read the journal’s policy and has the following potential conflicts: SP-P is a stockholder and the President and Chief Executive Officer of Somnogen Canada Inc., a Canadian Corporation. This does not alter his adherence to all of the journal policies. He declares that he has no competing interests that might be perceived to influence the content of this article. He further declares that he has no proprietary, financial, professional, nor any other personal interest of any nature or kind in any product or services and/or company that could be construed or considered to be a potential conflict of interest that might have influenced the views expressed in this manuscript.

